# Preparation and Properties of Na_2_HPO_4_∙12H_2_O/Silica Aerogel Composite Phase Change Materials for Building Energy Conservation

**DOI:** 10.3390/ma17215350

**Published:** 2024-10-31

**Authors:** Jiayue Lao, Jintao Ma, Zhili Zhao, Ning Xia, Jiesheng Liu, Hao Peng, Tao Fang, Wanwan Fu

**Affiliations:** School of Civil Engineering and Architecture, Wuhan Polytechnic University, Wuhan 430023, China; laojiayue0510@sina.com (J.L.); m1342264676@sina.com (J.M.); zhaozhili114@sina.com (Z.Z.); xianing9696@sina.com (N.X.); ljs628@whpu.edu.cn (J.L.); pengh@whpu.edu.cn (H.P.); tfang@whpu.edu.cn (T.F.)

**Keywords:** Na_2_HPO_4_∙12H_2_O, silica aerogel, composite phase change material, building energy conservation, thermal performance

## Abstract

In this paper, a morphologically stable composite phase change material (CPCM) suitable for use in the field of building energy conservation was developed using Na_2_HPO_4_∙12H_2_O (DHPD) as the phase change material, Na_2_SiO_3_∙9H_2_O (SSNH) as the nucleating agent, and silica aerogel (SA) as the carrier. The results showed that the incorporation of 25 wt% SA resulted in the as-prepared DHPD-SSNH/SA CPCM with a phase change temperature of 30.4 °C, an enthalpy of 163.4 J/g, and a low supercooling degree of 1.3 °C, which also solved the corrosion problem of reinforcing bars caused by the hydrated salt PCM. Meanwhile, DHPD-SSNH/SA CPCM had good shape stability and low thermal conductivity (0.1507 W/(m·K)). The phase change temperature was basically unchanged, and the enthalpy only decreased by 4.8% after 200 cold-heat cycles. In addition, the thermal performance evaluation of CPCM showed that the indoor thermal comfort time of the testing system loaded with PCM board accounted for 50.75%, which was 43.38% higher than that of the one without PCM board (7.37%). The results suggest that the obtained CPCM had a good energy saving effect and great potential in the field of building energy conservation.

## 1. Introduction

Building thermal insulation, as a passive thermal management method embedded in the building envelope, can reduce the effect of solar radiation on indoor temperatures and has received widespread attention in residential and commercial buildings and military applications [1]. By applying thermal insulation materials into the ceilings, walls, roofs, and windows of buildings [2], the method reduces the probability of using high-power equipment such as indoor air conditioning and heating and ventilation systems and therefore achieve high energy saving effects. In addition, it also has the advantages of simple installation, easy maintenance and replacement in the later stages, and high flexibility. In recent years, with environmental changes and the rapid growth of its population [3], the Earth’s temperature is rising, and hence traditional thermal insulation materials are increasingly unable to meet the current demand for temperature control effects. Meanwhile, a survey has shown that 80~90% of a person’s life is spent indoors [4], and this percentage in particular was as high as 100% during the coronavirus pandemic [5]. This exacerbates energy consumption in buildings, highlighting the growing gap between energy supply and consumption. In order to balance the mismatch between energy supply and consumption in buildings, researchers and engineers have applied phase change energy storage technology into buildings’ thermal insulation to develop building thermal insulation materials with a high thermal storage capacity and better temperature control effect [6].

The core of phase change energy storage technology is the phase change material (PCM), which can absorb or release a large amount of energy under almost isothermal conditions by utilizing its own phase change property [7]. Such a property of PCMs gives the technology a high energy storage density, small volume change, good safety, and controllable performance [8], being considered as the most promising technology at present. Therefore, the selection of a suitable PCM plays a pivotal role in improving the thermal insulation performance of buildings. Abden et al. [9] combined PCM board (paraffin RT-26) with traditional thermal insulation material (expanded polystyrene (EPS)) in the building envelope of a typical detached house in Australia and evaluated the thermal performance of the combined use with numerical simulations. The results showed that under the optimal combination of PCM board and EPS, significant cost savings could be achieved within 10 years of life, along with good building energy efficiency. Yasiri et al. [10] quantitatively studied the effects of different thicknesses of EPS on the thermal performance improvement of a building envelope with integrated paraffin PCM under severe summer conditions. The results showed that the maximum indoor temperature of the PCM-EPS room was reduced by 143%, the average indoor temperature fluctuation was reduced by 35%, and the average operating temperature was reduced by 8.5%, providing excellent thermal management performance. Huang et al. [11] prepared a novel composite PCM (CPCM) by combining a sodium acetate trihydrate (SAT)-formamide (FA) mixture with expanded perlite (EP) and introduced it into building roofs to optimize indoor thermal comfort. The results showed that the optimum mass fraction of EP adsorbable PCM was 55%, and the phase transition temperature of CPCM was 40.5 °C, with an enthalpy as high as 148 J/g and a low thermal conductivity of 0.0978 W/(m∙K). After testing, it was found that the temperature rate of the roof changed slowly, and the maximum temperature was reduced. Zeng et al. [12] used potassium chloride (KCl) as a thermoregulating agent, strontium hydroxide octahydrate (Sr(OH)_2_∙8H_2_O) as a nucleating agent, and expanded graphite (EG) as a carrier to prepare CPCM with a phase transition temperature of 26.25 °C, enthalpy of 153.03 J/g, and thermal conductivity of 4.3893 W/(m∙K) using a physical mixing method. Through the thermoregulation performance comparisons with the polyvinyl chloride (PVC) sheet composites for temperature regulation performance comparison, it was found that the chamber temperature of the PCM model was maintained at 24.5–27.5 °C for as long as 652 min during the heating and cooling process, having a good temperature control effect. The studies above proved that the integration of PCM into a building envelope can optimize the heat transfer characteristics of the envelope and improve indoor thermal comfort, thereby realizing a good energy saving effect. Therefore, the Na_2_HPO_4_∙12H_2_O (DHPD)-Na_2_SiO_3_∙9H_2_O (SSNH) mixture with a suitable phase transition temperature (35.3 °C) and high enthalpy (215.3 J/g) which was previously developed [13] was used as the PCM in the present study. However, the actual engineering application proved that a single DHPD-SSNH PCM is prone to leakage, which leads to a serious drop in enthalpy, reduces energy saving, and causes serious corrosion to reinforcing bars as well. Therefore, the DHPD-SSNH PCM needs to be wrapped or shaped prior to practical application to solve the DHPD-SSNH PCM leakage problem.

Currently, the commonly used method is the porous adsorption method [14,15], which utilizes hydrogen bonding, capillary force, and surface tension to adsorb and immobilize the PCM in the pore structure of a porous carrier, such as silica aerogel (SA) [16] or expanded graphite (EG) [17]. The as-prepared material is regarded as a CPCM. Among them, SA consists of microscopic amorphous silica in chains [18] aggregated into a porous three-dimensional mesh carrier. It is an ideal lightweight porous matrix with a strong adsorption capacity, low thermal conductivity, high specific surface area, and high porosity which is non-toxic and fire-resistant [19]. Zhang et al. [20] used SA as a carrier and octacosanol as a PCM to prepare a CPCM, which had thermal conductivity of 0.0277 W/(m∙k), a large energy storage capacity, and an excellent thermal insulation property. Cao et al. [21] used methyl palmitate, disodium hydrogen phosphate dodecahydrate, and sodium carbonate decahydrate as a three-phase PCM, sodium carboxymethyl cellulose as a thickener, and SA as a porous carrier, and they successfully prepared a new type of shape-stabilized organic/inorganic CPCM for thermal management of buildings. Shen [22] et al. used SA to adsorb paraffin and form CPCM, which was then blended into cement to prepare thermal storage cement board for comparative experiments. The results showed that during cooling and heating, the maximum temperature difference of 10 mm-thick thermal storage cement board was 2.36 °C and 1.92 °C, respectively, compared with ordinary cement board, showing a good thermal insulation effect. Arshad et al. [23] used polyethylene glycol (PEG) as PCM and SA as an adsorption matrix to prepare PEG/SA CPCM as an energy-efficient material for buildings. The results showed that PEG/SA CPCM loaded into a building’s structure reduced the indoor temperature by about 20%, and the temperature difference between outdoor and indoor weather conditions was up to about 20 °C, providing good temperature control performance. The results showed that the prepared CPCM exhibited excellent shape stability and flame retardance and had no subcooling or phase separation problems. Meanwhile, it had a high latent heat value (174.1 J/g) and a suitable phase transition temperature (22.9 °C), and its thermal properties remained basically unchanged after 100 cold-heat cycles. It can be seen from the research above that by using SA as carrier, its low thermal conductivity and excellent adsorption performance gave the resulting CPCM excellent thermal performance, exhibiting great potential for application in the field of building energy conservation.

Up to now, the application of CPCM in the field of building energy conservation has attracted the interest of many researchers. However, the connection between DHPD-SSNH PCM and SA has not yet been established, and the application in the field of building energy conservation is still unknown, which has a great influence on the development of DHPD-SSNH PCM. This makes the related research work valuable. Therefore, on the basis of the above studies, and considering the excellent properties of SA, a new DHPD-SSNH/SA CPCM with high enthalpy was prepared in this study by using SA as the porous carrier and a DHPD-SSNH mixture as the PCM with the physical mixing method. The CPCM can be either integrated into a building envelope as a PCM board or treated as a fine aggregate by combining it with the building materials and integrating it into the interior of building structures for multi-scenario applications. In this research work, leakage experiments, experiments on the corrosion of reinforcing bars, N_2_ adsorption and desorption, and SEM characterization revealed that the porous SA had a strong adsorption capacity, which can solve the problem of PCM leakage. FT−IR and XRD characterization showed that the mixing between DHPD-SSNH PCM and SA was only physical. Meanwhile, the CPCM had extremely low thermal conductivity and excellent thermal reliability. In addition, in this study, the CPCM was made into PCM board, and a thermal performance evaluation system was designed to simulate the effect of PCM panels embedded in a building envelope on the indoor environment to examine the thermal insulation performance of CPCM and explore the possibility of its application in building energy conservation.

## 2. Experiment

### 2.1. Materials

Disodium hydrogen phosphate dodecahydrate (DHPD) (Na_2_HPO_4_∙12H_2_O, AR) and sodium silicate hydrate (SSNH) (Na_2_SiO_3_∙9H_2_O, AR) were commercially purchased from Shanghai Sinopharm Chemical Reagent Co., Ltd. in China and the Ningbo Tailande Chemical Reagent Factory in China, respectively. Silica aerogel (SiO_2_, SA, hydrophilic) was provided by Suzhou Kangmai New Material Co., Ltd. in China. The polycarbonate hollow plate was provided by Suzhou Bakway New Material Co., Ltd. in China.

### 2.2. Preparation of DHPD-SSNH/SA CPCM

**Figure 1:**
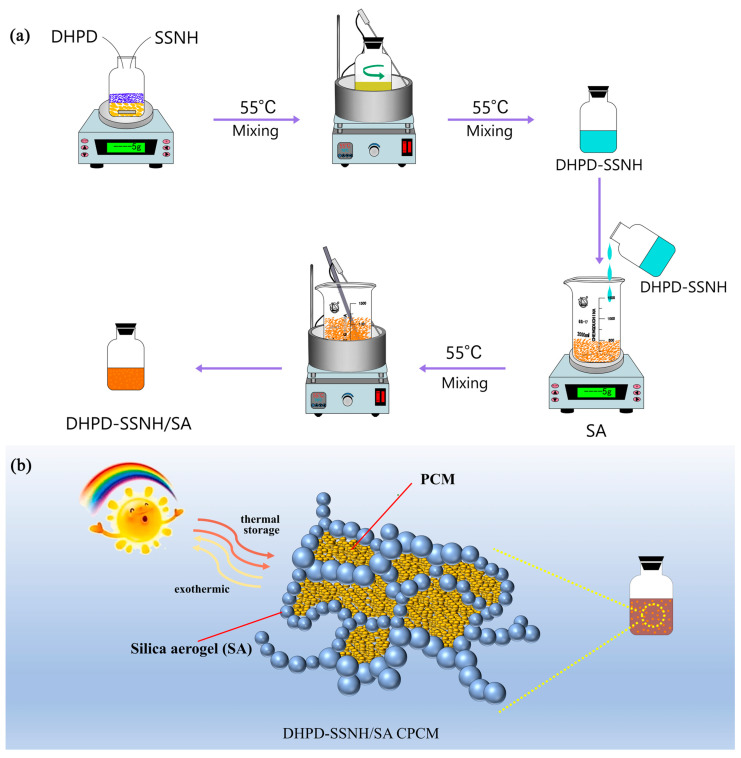
Preparation process (**a**) and work schematic (**b**) of CPCM.

Figure 1 shows the preparation process of DHPD-SSNH/SA CPCM containing different mass fractions of SA. Firstly, before compositing, the SA was placed in an oven and dried at 110 °C for 2 h to remove the water inside the pores and set aside. Subsequently, 30 g DHPD and 1.2 g SSNH were packed into a clean reagent bottle and stirred in a magnetic stirring pot at 55 °C until all materials melted (i.e., DHPD-SSNH PCM was obtained). Then, the obtained PCM was added dropwise to a beaker containing different mass ratios (15 wt%, 20 wt%, 25 wt%, 30 wt%, and 35 wt%) of SA and stirred with a glass rod at room temperature for 10 min. After stirring, the beaker was completely sealed and heated in a thermostatic water bath at 55 °C for 60 min. The heating-stirring process was repeated 3 times for the SA to naturally adsorb the PCM and obtain the DHPD-SSNH/SA CPCM.

### 2.3. Characterization

The test method for the supercooling degree was as follows. First, 25 g of DHPD-SSNH/SA CPCM was loaded into a reagent bottle, inserted into a type K thermocouple, and heated to a constant temperature in an oven at 50 °C. The instrument was then switched off and cooled to room temperature (approximately 20 °C), and the temperature changes throughout the process were recorded using a data acquisition instrument. The thermal reliability of the DHPD-SSNH/SA CPCM was tested in a constant temperature in a humidity chamber (Zhong Ya, 512b, China) at 10~60 °C for 200 cycles.

The pore structures of the SA and DHPD-SSNH/SA CPCM were analyzed using an pore size analyzer (Autosorb iQ-MP, Boynton Beach, FL, USA). The micro-morphologies of the SA and DHPD-SSNH/SA CPCM were observed using a model 8100 scanning electron microscope (SEM, Tokyo, Japan), and the elemental distribution of the CPCM was analyzed with an energy spectrum analyzer (EDS, Tokyo, Japan). X-ray diffractometry (Smartlab SE, Tokyo, Japan) was used to analyze the crystalline structures of the materials in the scanning range of 5~80°. An FT-IR spectrometer (Bruker, Karlsruhe Germany) was used to analyze the chemical compositions of the materials in the range of 4000~600 cm^−1^. The thermal conductivity of the prepared materials was measured with a thermal constant analyzer (TPS2500, Gothenburg, Sweden), and the heat transfer process was observed with a infrared thermal imager (K20, Shenzhen, Chinese). The phase transition parameters of the samples were recorded with a model 300 differential scanning calorimeter (DSC) (Netzsch, Bavaria, Germany).

The theoretical enthalpies of the phase transitions of the CPCMs were calculated and determined using Equation (1) [24]:

∆*H*_m,theory_ = ∆*H*_m,PCM_ × (1 − ω_SA_) (1)

where ∆*H*_m,PCM_ is the actual phase change enthalpy of the DHPD-SSNH PCM in Joules per gram and ω_SA_ is the percentage of SA in the CPCM.

### 2.4. Effect of DHPD-SSNH/SA CPCM on Thermal Performance of Buildings

**Figure 2:**
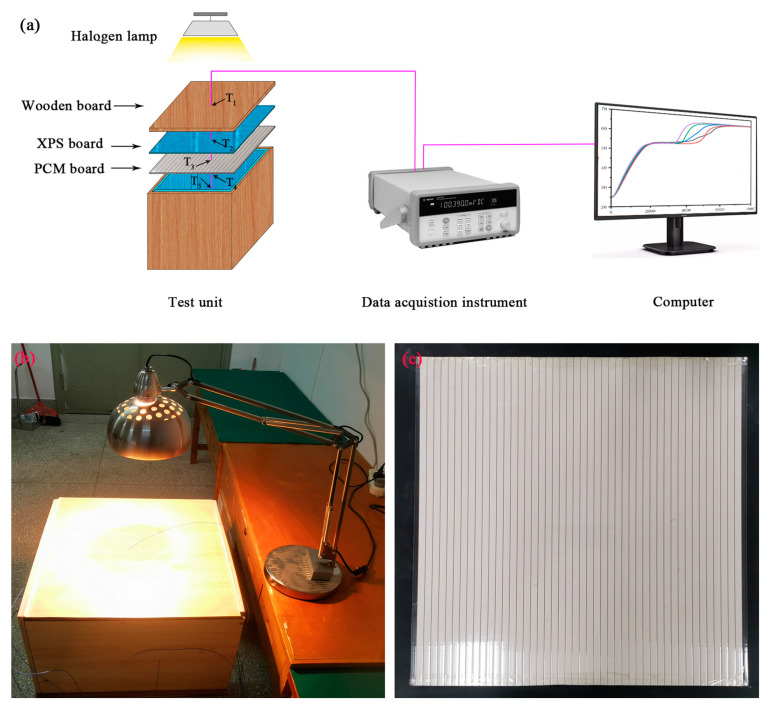
Flowchart of the 24 h simulation test system (**a**), actual pictures of the model (**b**), and PCM board (**c**).

In order to assess the energy saving effect of the prepared DHPD-SSNH/SA CPCM in building thermal management, a 24 h simulation test system was constructed at an indoor ambient temperature of 20 °C, as shown in Figure 2. The constructed test system consisted of four parts—a test unit, a data acquisition instrument, a computer, and a heating unit—as shown in Figure 2a. Among these, the DHPD-SSNH/SA CPCM was packed into a polycarbonate hollow plate to prepare a PCM board with a thickness of 10 mm, and then it was tightly combined with an XPS plate (20 mm thick) and a wood plate (15 mm thick) in order to form a test unit of 500 × 500 × 300 mm (L × W × H). The heating unit used a 275 W-ZJD-10 infrared baking lamp placed about 165 mm above the test unit to simulate daylight irradiation and provide heat for the test unit. At the same time, a temperature probe was set in the center of the test unit’s room and at the junction of each layer of the structure, which recorded the temperature changes of the heating unit when turned on or off for 12 h.

## 3. Results and Discussion

### 3.1. Optimal Loading of DHPD-SSNH PCM with SA

**Figure 3:**
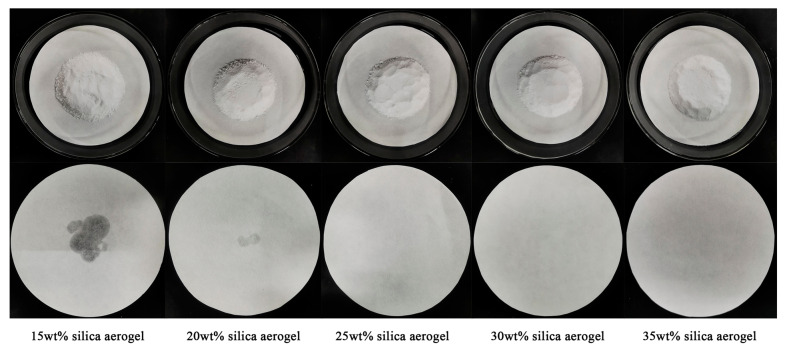
Loading conditions of DHPD-SSNH PCM with different mass fractions of SA.

In this section, the leakage conditions of DHPH-SSNH/SA CPCMs containing different mass fractions of SA (15 wt%, 20 wt%, 25 wt%, 30 wt%, and 35 wt%) on filter papers were observed with a leaking test as a method to assess the loading effect of the SA adsorption matrix on the DHPD-SSNH PCM (refer to Figure 3). After the heat treatment, a large area of liquid DHPD-SSNH PCM leakage appeared on the DHPH-SSNH/SA CPCM filter paper with an SA mass fraction of 15%, and a small amount of liquid also appeared on the filter paper with an SA mass fraction of 20%, indicating that the 15 wt% and 20 wt% SA were not fully loaded onto the corresponding mass fractions of the DHPD-SSNH PCM. In contrast, the DHPD-SSNH/SA CPCM did not show leakage, as can be seen on the 25 wt%, 30 wt%, and 35 wt% SA filter papers. This indicates that SA could adsorb the DHPD-SSNH PCM through surface tension and capillary force when the SA mass fraction was 25% or more, as well as the formation of a CPCM with good shape stability. Therefore, the DHPH-SSNH/SA CPCMs with SA mass fractions of 25%, 30%, and 35% were selected for further study.

### 3.2. Effect of SA Mass Fraction on Thermal Properties

**Figure 4:**
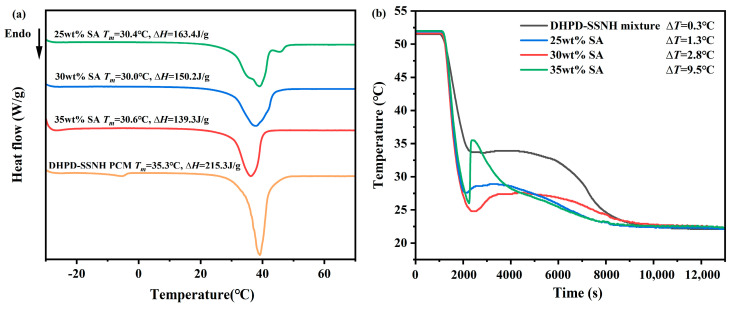
DSC curves (**a**) and step cooling curves (**b**) of CPCMs with different ratios of SA.

Based on the above leakage test results, in order to reveal the influence of the SA mass fraction on the phase transition temperature (*T_m_*) and the enthalpy of the phase transition (∆*H_m_*) of DHPD-SSNH/SA CPCMs, tests were carried out using DSC. Figure 4a shows the DSC curves of DHPH-SSNH/SA CPCMs with SA mass fractions of 25%, 30%, and 35%, and the results are shown in Table 1.

Figure 4a and Table 1 show that the *T_m_* of the DHPD-SSNH PCM was 35.3 °C and the ∆*H_m_* was 215.3 J/g. When 25%, 30% and 35% SA were added, the *T_m_* of the CPCMs reduced to around 30 °C for the confinement effect of pores originating from the SA and the interactions between the PCM and SA. The tested values for the ∆*H_m_* were in the range of 139.3~163.4 J/g and showed a proportional decreasing trend, due to the fact that SA did not provide the corresponding enthalpy value in the testing temperature range of the DHPH-SSNH/SA CPCM, resulting in a decrease in the ∆*H_m_* for the CPCMs with increasing SA mass fractions. The relative errors of the ∆*H_m,theory_*, calculated according to Equation (1), were controlled within 1.18% compared with the actual tested ∆*H_m_*, indicating that the measured ∆*H_m_* values agreed with the ∆*H_m,theory_*.

The supercooling degree (∆*T*) of the DHPH-SSNH/SA CPCMs with SA mass fractions of 25%, 30%, and 35% was tested, and the results are shown in Figure 4b. The ∆*T* of the DHPD-SSNH PCM was 0.3 °C, being 1.3 °C, 2.8 °C, and 9.5 °C for DHPH-SSNH/SA CPCMs with 25 wt%, 30 wt%, and 35 wt% SA added, respectfully. Clearly, the ∆*T* became larger with the rise in the proportion of SA, probably because the addition of a mass of SA impeded the formation and development of DHPD-SSNH PCM nuclei, resulting in the increase in ∆*T* [25]. Therefore, considering the results of the loading effect, DSC, and ∆*T*, the SA mass fraction of 25% was selected as the optimal ratio for loading the DHPD-SSNH PCM, and the next study was carried out.

### 3.3. Corrosion Analysis of DHPD-SSNH/SA CPCM on Reinforcing Bars

**Figure 5:**
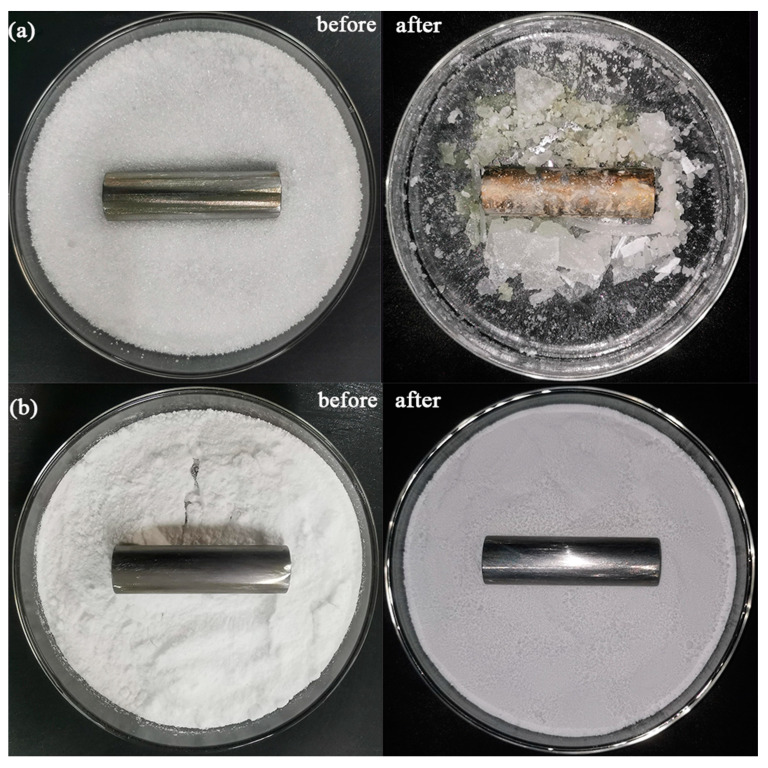
Corrosion of reinforcing bars in pure DHPD (**a**) and CPCM (**b**).

Non-corrosivity is an important property for the development of DHPD-SSNH/SA CPCM in the construction field [26]. Therefore, in order to test whether the SA adsorption matrix could solve the corrosion problem of DHPD-SSNH PCM due to leakage of the steel reinforcement inside buildings, two polished HPB300 steel bars were fully coated with DHPD and CPCM, respectively, and then heated up in an oven at 60 °C for 7 days, as shown in Figure 5. As can be seen in Figure 5a,b, the reinforcing bars immersed in the CPCM did not have any obvious signs of corrosion or corrosion products, while the one immersed in DHPD was seriously rusted. This phenomenon may be induced by the melting of the heated DHPD. On one hand, the generated phosphate ions (HPO_4_^2−^) formed an electrolyte layer on the surface of the reinforcing bar in its aqueous solution, which underwent an electrochemical reaction and accelerated the corrosion. On the other hand, although the HPO_4_^2−^ itself did not directly cause severe pitting corrosion like the chloride ion, it may have changed the local pH value and affected the passivation layer of the reinforcing bar, exposing the reinforcing bar to a corrosive environment. As for the PCM after being adsorbed and shaped by the SA, the DHPS-SSNH PCM was firmly adsorbed in the pore structure via the surface tension and capillary force, avoiding the leakage problem and not causing the corrosion phenomenon on the reinforcing bar. Accordingly, the DHPD-SSNH/SA CPCM with an SA mass fraction of 25% could effectively solve the corrosion problem of hydrated salt-based PCM on reinforcing bars when applied to construction projects.

### 3.4. Pore Structure Analysis

**Figure 6:**
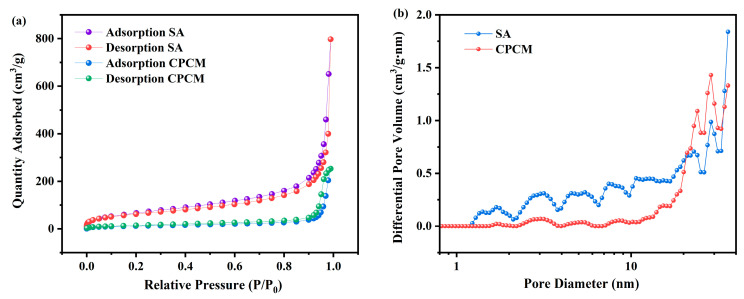
N_2_ adsorption-desorption isotherms (**a**) and pore size distributions (**b**) of SA and CPCM.

The N_2_ adsorption-desorption isotherms and pore size distributions of the SA and CPCM are shown in Figure 6, and the results for the specific surface area (S_BET_) and pore volume (V) are shown in Table 2. It can be seen from Figure 6a that the SA exhibited type IV isotherms after the adsorption-desorption of N_2_, which suggests that mesopores were present in the pore structure of the SA [27]. The pore size distribution curve in Figure 6b further confirms the above result that the pore structure of the SA was mainly composed of mesopores in the range of 2.7~30 nm. In addition, for the test results in Table 2, the SA existed large S_BET_ (224.508 m^2^/g) and V values (0.395 cm^3^/g), which can provide a strong loading capacity. When the DHPD-SSNH PCM was adsorbed by the SA, the S_BET_ and V values of the CPCM decreased to 8.68 m^2^/g and 0.108 cm^3^/g, respectively, and the loading capacity was significantly reduced. This indicates that the DHPD-SSNH PCM was successfully adsorbed by the SA and stereotyped. Subsequent SEM microstructure analysis further verified this result.

### 3.5. Micromorphology Analysis

**Figure 7:**
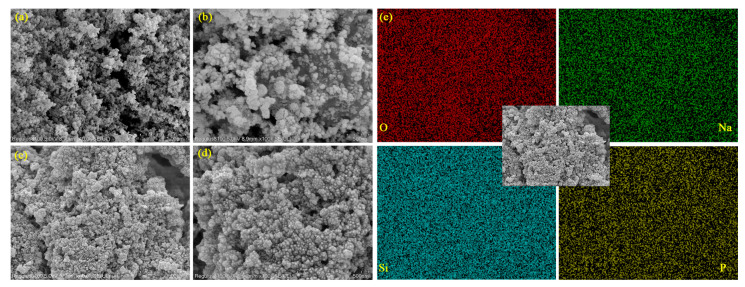
SEM images of SA (**a**,**b**) and CPCM (**c**,**d**) at 40,000× and 100,000× magnification, respectively, with EDS images of elements O, Na, Si, and P (**e**).

The micro-morphologies of the SA and DHPD-SSNH/SA CPCM were observed and analyzed using SEM, as seen in Figure 7. To fully observe the microstructure of the SA and the adsorption condition of the PCM, magnifications of 40,000× ((a) and (c)) and 100,000× ((b) and (d)) were selected for the observation and analysis. As can be seen in Figure 7a,b, the SiO_2_ nanoparticles were densely cross-linked to form a quite obvious porous, three-dimensional (3D) mesh structure, giving it a strong adsorption capacity. It can also be clearly seen in Figure 7c,d that after the DHPD-SSNH PCM was composited with the SA, the DHPD-SSNH PCM was adsorbed into the porous 3D mesh pores and adhered to the surfaces of the SiO_2_ nanoparticles under the actions of capillary force and surface tension, which prevented the PCM from leaking under high-temperature operation.

In addition, to show the distribution of the DHPD-SSNH PCM in the CPCM more clearly, the sample was analyzed with an energy spectrum, as shown in Figure 7e. From the elemental distribution in Figure 7e, it is obvious that the elements O, Na, Si, and P, which correlated with the constitution of the PCM, were uniformly distributed on the surface of the SA, indicating that the DHPD-SSNH PCM was successfully loaded onto the SA adsorption matrix. It was further verified that SA is an ideal carrier for solving the leakage problem of DHPD-SSNH PCM.

### 3.6. Chemical Composition and Crystal Structure

**Figure 8:**
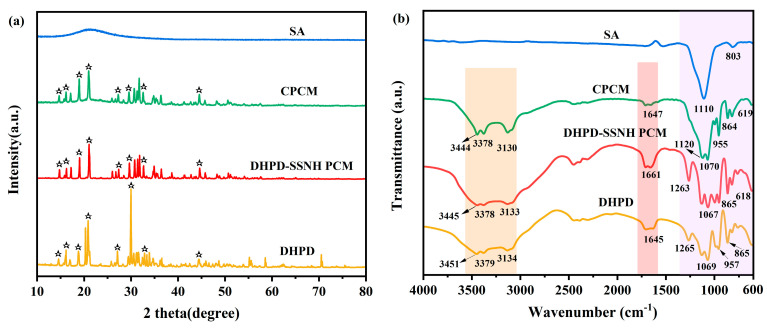
XRD patterns (**a**) and FT−IR spectra (**b**) of hydrated salts, SA, and CPCM.

The XRD patterns of the hydrated salts, CPCM, and SA are shown in Figure 8. In Figure 8a, it can be seen that there was no obvious diffraction peak for the SA, indicating that the SA was amorphous [28]. Meanwhile, in the XRD pattern of the DHPD-SSNH PCM, obvious diffraction peaks were observed at 2θ values of 14.58°, 16.16°, 18.80°, 20.86°, 27.10°, 29.98°, 32.86°, and 44.42°, all of which could be found in the patterns of the CPCM, and no new diffraction peaks appeared. This indicates that there was a physical combination between the SA and PCM without a chemical reaction.

Figure 8b shows the FT−IR spectra of the prepared and raw materials. It is noted from Figure 8b that the FT−IR spectrum of the CPCM was a combination of those of the DHPD-SSNH PCM and SA. Meanwhile, no new peaks appeared, indicating that no chemical interaction between the SA and PCM occurred. It is noteworthy that there was a broader peak in the 2750–3750 cm^−1^ interval of the FT−TR spectra of the DHPD, DHPD-SSNH PCM, and CPCM, which belonged to the telescopic vibration peak of the O-H group. There were also obvious absorption peaks at 1110 cm^−1^ and 803 cm^−1^ in the FT−IR spectrum of the SA, which belonged to the stretching vibration peak and bending vibration peak of Si–O–Si, respectively. Therefore, the XRD and FT−IR results indicate that the bonding between the SA and PCM was physical in nature with no chemical reaction.

### 3.7. Thermal Conductivity

**Figure 9:**
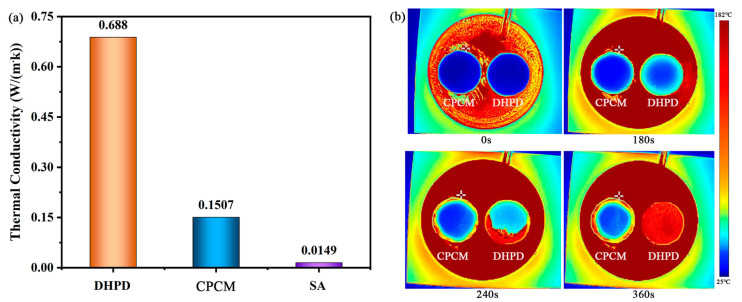
Thermal conductivity of the material (**a**) and infrared thermograms at 0 s, 180 s, 360 s, and 480 s (**b**).

By optimizing the thermal performance of the building envelope, the heat transfer characteristics of the building can be altered, and the indoor thermal comfort is accordingly improved. In Figure 9a, SA possesses rather low thermal conductivity (0.0149 W/(m·K)). Its good thermal insulation property makes it block part of the heat from the Sun’s thermal radiation to the building in the peripheral environment, and it maintains comfortable indoor temperatures for a long time. The thermal conductivity of DHPD was 0.688 W/(m·K), and after being adsorbed by SA to form the DHPD-SSNH/SA CPCM, the coefficient of thermal conductivity was reduced to 0.1507 W/(m·K). Although the thermal conductivity of the DHPD-SSNH/SA CPCM was 10 times higher than that of SA, it was still at a lower level, making it ideal for use from the aspect of building energy conservation.

The heating processes of CPCM and DHPD were observed and analyzed using infrared thermography, as shown in Figure 9b. When DHPD and CPCM were placed in a high-temperature environment at the same time, the DHPD was heated and melted rapidly, melting completely in 360 s. The temperature of the CPCM changed slowly from the edge to its center, but the rate of temperature change was always lower than that of DHPD. This was mainly because the CPCM had low thermal conductivity, thereby blocking the heat transfer and showing strong thermal inertia. Therefore, the CPCM prepared in this work had good thermal insulating properties and also verified the results of the thermal conductivity mentioned above.

### 3.8. Thermal Reliability

**Figure 10:**
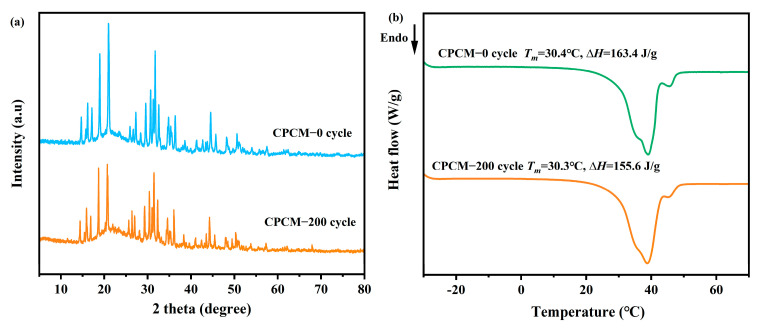
XRD spectra (**a**) and DSC curves (**b**) of CPCM before and after 200 cycles.

The CPCM must have good thermal reliability to keep its performance unchanged in practical engineering applications. Therefore, the DHPD-SSNH/SA CPCM was put into a constant temperature and humidity chamber for 200 cold-heat cycles, and the thermal properties and crystal structures of the sample after cycling were characterized and tested using DSC and XRD, as shown in Figure 10. In Figure 10a, after 200 cycles, the positions of the diffraction peaks of the DHPD-SSNH/SA CPCM correspond to those of the material before cycling, and meanwhile, the intensities were almost unchanged, indicating that the CPCM had excellent stability of the crystal structure. In Figure 10b, the *T_m_* was 30.3 °C, and the ∆*H_m_* was 155.6 J/g for the CPCM after 200 cycles. Compared with the sample before cycling, the *T*_m_ was almost unchanged, and the loss for the ∆*H_m_* was 4.8%. The loss in the ∆*H_m_*, which was probably caused by evaporation of water in the crystallization of the DHPD-SSNH PCM, was still within the acceptable range. The excellent thermal reliability of DHPD-SSNH/SA CPCM could help maintain good working performance for a long period of time in practical engineering applications.

### 3.9. Assessment of Impact of CPCM on Thermal Performance of Buildings

**Figure 11:**
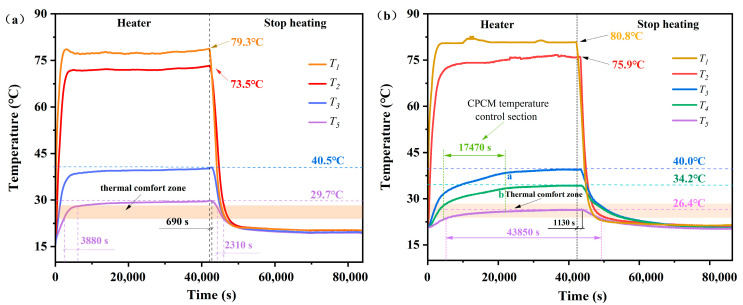
Temperature variation curves of test systems without PCM board (**a**) and with PCM board (**b**).

In a previous study [13], a thermal performance evaluation system was constructed to test the percentage of thermal comfort time in the room of a testing system when no PCM board was mounted, as shown in Figure 11a. Therefore, using the previous research as a reference, the DHPD-SSNH/SA CPCM prepared in this work was fabricated into PCM board and loaded into a test system to form a test unit. The temperature changes in the center of the room and at the junction of each structural layer were tested to evaluate the thermal performance of the DHPD-SSNH/SA CPCM in terms of the percentage of time taken for thermal comfort [11]. The testing schematic diagram is presented in Figure 2.

The temperature-versus-time curves of the testing system with PCM board are shown in Figure 10b. From Figure 10b, it can be seen that the temperature of the outer surface of the testing system (*T*_1_) and the outer surface of the XPS (*T*_2_) increased rapidly under heating of the infrared baking lamp and tended to equilibrate around 80.8 °C and 75.9 °C, respectively. It is worth noting that the temperature curves of the outer surface (*T*_3_) and the inner surface (*T*_4_) of the PCM board had turning points at points a and b. The temperature control time was as long as 17,470 s, which accounted for 40.44% of the heating time (12 h), and then tended to equilibrate near 40.0 °C and 34.2 °C, respectively. This phenomenon was mainly caused by absorbing part of the heat during the solid-to-liquid transition of the PCM when the ambient temperature was higher than the *T_m_* of the DHPD-SSNH/SA CPCM. This indicates that the prepared DHPD-SSNH/SA CPCM had good temperature control performance.

Compared with other locations, the center temperature (*T*_5_) of the testing system with the CPCM board changed more slowly over time. According to the Design Code for Heating Ventilation and Air Conditioning of Civil Buildings [29], which has been implemented since 1 October 2012, the indoor thermal comfort temperature should be at 24~28 °C in summer. Therefore, it can be seen from Figure 10b that the time when *T*_5_ was within the range of 24~28 °C was 43,850 s, accounting for 50.75% of the total time of (24 h). In contrast, in Figure 10a, the thermal comfort time of *T_5_* in the testing system without PCM board was 6190 s, which was only 7.37% of the total time. In addition, with the data in Table 3, in a previous study, the energy-saving effect of the PCM panels with diatomite as a carrier in the previous research work was 40.09%, while the PCM panels with SA as a carrier in this study had better performance, with 43.38% energy saving. It is worth noting that the DHPD-SSNH/SA CPCM had lower thermal conductivity and better thermal insulation performance than the DHPD-SSNH/diatomite CPCM. The results show that the DHPD-SSNH/SA CPCM had excellent energy-saving effects, and it is suitable for use in buildings.

## 4. Conclusions

In this paper, a novel, morphologically stabilized DHPD-SSNH/SA CPCM was synthesized, using a DHPD-SSNH mixture as the PCM and SA as the carrier, for thermal conservation in buildings. Its performance was analyzed, and the following research conclusions were drawn:

(1) When adding 25% SA, the prepared DHPD-SSNH/SA CPCM had good shape stability, with a *T_m_* of 30.4 °C, a ∆*H_m_* of 163.4 J/g, thermal conductivity of 0.1507 W/(m·K), and a low supercooling degree of 1.3 °C.
(2) The SEM and pore structure analysis results show that the DHPD-SSNH PCM was confined in the three-dimensional mesh structure of SA, which could effectively inhibit the leakage of PCMs. The FT-IR and XRD results indicated that the SA and PCM in the DHPD-SSNH/SA CPCM were physically bonded and did not involve chemical reactions.
(3) Corrosion experiments showed that the DHPD-SSNH/SA CPCM with 25% SA could effectively solve the corrosion problem of hydrated, salt-based PCMs in reinforcing bars in practical applications.
(4) After 200 cold-heat cycles, the *T_m_* of CPCM was almost unchanged, and the loss of ∆*H_m_* was only 4.8%, with the unchanged crystal structure and chemical composition possessing good thermal reliability.
(5) The thermal performance evaluation showed that the PCM board was equipped with an excellent energy-saving effect by increasing the thermal comfort time in the center of the test unit room by 43.38%.

## Figures and Tables

**Table 1:** Phase transition parameters of CPCMs with different ratios of SA.

Mass Fraction of SA (%)	*T_m_* (°C)	∆*H_m_* (J/g)	∆*H_m_*_,*theory*_ (J/g)	Relative Errors (%)	∆*T* (°C)
0	35.3	215.3	-	-	0.3
25	30.4	163.4	161.5	−1.18	1.3
30	30.0	150.2	150.7	0.33	2.8
35	30.6	139.3	139.9	0.43	9.5

**Table 2:** Test results for pore structure parameters.

Sample	S_BET_ (m^2^/g)	V (cm^3^/g)
SA	224.508	0.395
DHPD-SSNH/SA CPCM	43.650	0.108

**Table 3:** Comparison of energy savings between different test units.

Sample	Thermal Conductivity (W/(m·K))	Thermal Comfort Time (s)	Percentage of Thermal Comfort Time (%)	Energy-Saving Effect (%)
Reference group	-	6190	7.37	-
DHPD-SSNH/diatomite CPCM [13]	0.2400	39,870	47.46	40.09
DHPD-SSNH/SA CPCM	0.1507	43,850	50.75	43.38

## Data Availability

The original contributions presented in the study are included in the article, further inquiries can be directed to the corresponding author.

## Nomenclature

*Parameters*	
*T_m_*	Phase change temperature (°C)
∆*H_m_*	Measured phase change enthalpy (J/g)
∆*H*_*m*,*theory*_	Theoretical phase change enthalpy (J/g)
∆*T*	Supercooling degree (°C)
*T*_1_	Temperature of the outer surface of the test unit (°C)
*T*_2_	XPS outer surface or wood plate inner surface temperature (°C)
*T*_3_	PCM outer surface or XPS inner surface temperature (°C)
*T*_4_	PCM plate internal surface temperature (°C)
*T*_5_	Room center temperature (°C)
S_BET_	Surface area (m^2^/g)
V	Pore volume (cm^3^/g)
*Abbreviations*	
PCM	Phase change material
CPCM	Composite phase change materials
XRD	X-ray diffraction
FT-IR	Fourier transform infrared
SEM	Scanning electron microscope
DSC	Differential scanning calorimetry
BET	Brunauer–Emmett–Teller
DFT	Density functional theory
